# A single codon insertion in PICALM is associated with development of familial subvalvular aortic stenosis in Newfoundland dogs

**DOI:** 10.1007/s00439-014-1454-0

**Published:** 2014-06-05

**Authors:** Joshua A. Stern, Stephen N. White, Linda B. Lehmkuhl, Yamir Reina-Doreste, Jordan L. Ferguson, Nanette M. Nascone-Yoder, Kathryn M. Meurs

**Affiliations:** 1 Department of Clinical Sciences College of Veterinary Medicine, North Carolina State University, Raleigh, NC 27607 USA; 2 Department of Veterinary Clinical Sciences, College of Veterinary Medicine, Washington State University, Pullman, WA 99164 USA; 3 Department of Medicine and Epidemiology, School of Veterinary Medicine, University of California Davis, 2108 Tupper Hall, One Shields Ave, Davis, CA 95616 USA; 4 Animal Disease Research Unit, Agricultural Research Service, U.S. Department of Agriculture, Pullman, WA 991644 USA; 5 Department of Veterinary Microbiology and Pathology, Washington State University, Pullman, WA 99164 USA; 6 MedVet Medical and Cancer Centers for Pets, Worthington, OH 43085 USA; 7 Department of Molecular Biomedical Sciences, College of Veterinary Medicine, North Carolina State University, Raleigh, NC 27607 USA

## Abstract

Familial subvalvular aortic stenosis (SAS) is one of the most common congenital heart defects in dogs and is an inherited defect of Newfoundlands, golden retrievers and human children. Although SAS is known to be inherited, specific genes involved in Newfoundlands with SAS have not been defined. We hypothesized that SAS in Newfoundlands is inherited in an autosomal dominant pattern and caused by a single genetic variant. We studied 93 prospectively recruited Newfoundland dogs, and 180 control dogs of 30 breeds. By providing cardiac screening evaluations for Newfoundlands we conducted a pedigree evaluation, genome-wide association study and RNA sequence analysis to identify a proposed pattern of inheritance and genetic loci associated with the development of SAS. We identified a three-nucleotide exonic insertion in phosphatidylinositol-binding clathrin assembly protein (PICALM) that is associated with the development of SAS in Newfoundlands. Pedigree evaluation best supported an autosomal dominant pattern of inheritance and provided evidence that equivocally affected individuals may pass on SAS in their progeny. Immunohistochemistry demonstrated the presence of PICALM in the canine myocardium and area of the subvalvular ridge. Additionally, small molecule inhibition of clathrin-mediated endocytosis resulted in developmental abnormalities within the outflow tract (OFT) of *Xenopus laevis* embryos. The ability to test for presence of this PICALM insertion may impact dog-breeding decisions and facilitate reduction of SAS disease prevalence in Newfoundland dogs. Understanding the role of PICALM in OFT development may aid in future molecular and genetic investigations into other congenital heart defects of various species.

## Introduction

Subvalvular aortic stenosis (SAS) is one of the most commonly reported congenital heart defects in dogs (Buchanan 1999; Tidholm 1997). It is characterized by an abnormal ridge or ring of tissue in the left ventricular outflow tract (LVOT) that resists ventricular ejection, generates pressure overload, and increases velocity of blood flow into the aorta (Pyle and Patterson 1976; Jones et al. 1982). The gold standard for diagnosis of SAS is the demonstration of a subvalvular ridge or ring on post-mortem examination. Antemortem diagnosis is conventionally established by increased LVOT velocity reported by spectral Doppler echocardiogram studies and is augmented by the presence of supportive findings such as presence of a visible subvalvular ridge, left ventricular hypertrophy, post-stenotic aortic dilation and aortic insufficiency (O’Grady et al. 1989).

Although dogs with a mild form of the disease may have a normal lifespan, severely affected dogs may experience life-threatening arrhythmias, congestive heart failure, endocarditis and sudden death. Average lifespan for dogs with severe SAS in one study was just 19 months (Kienle et al. 1994). With medical therapy generally consisting of beta-blockade, SAS-affected dogs live an average of 4.5 years. Although interventional and surgical techniques have been evaluated for treatment of SAS, no study has shown any long-term benefit to these approaches that exceeds traditional medical therapy (Meurs et al. 2005). This observation has led to an increased interest in disease prevention through a heightened understanding of the disease etiology.

Subvalvular aortic stenosis is known to be an inherited defect in Newfoundland dogs, golden retrievers and children (Pyle and Patterson 1976; Jones et al. 1982; Stern et al. 2012; Petsas et al. 1998; Wessels et al. 2009). The pattern of inheritance in Newfoundland dogs was previously investigated in a single extended family of dogs and demonstrated to be either autosomal dominant with incomplete penetrance or polygenic in origin (Pyle and Patterson 1976). To our knowledge, molecular analysis of this disease in Newfoundland dogs has never been reported. The objective of this study was to evaluate the familial nature of SAS in the Newfoundland through pedigree analysis and genome-wide association.

## Materials and methods

This study was conducted under the guidelines of the Animal Care and Use Committees of Ohio State University, Washington State University and North Carolina State University.

SAS-affected and unaffected Newfoundland dogs were recruited for participation in a study to investigate the genetic aspects of SAS in this breed. Dogs were evaluated by veterinary cardiologists at two veterinary teaching hospitals in the United States of America. Cardiac auscultation and routine echocardiogram were performed on each animal. Pedigree information and a DNA sample were collected.

Two-dimensional echocardiograph including Doppler evaluations were performed by board certified cardiologists or cardiology residents in training. Maximal aortic outflow tract velocity (LVOT *V*_max_) was obtained from subcostal imaging, continuous-wave, spectral Doppler tracings during normal sinus rhythm. The diagnosis of SAS was made by combined auscultation and echocardiography. Guidelines for distinguishing SAS-affected dogs from normal dogs developed by the cardiology diplomates of the American College of Veterinary Internal Medicine stipulate LVOT maximal velocities (*V*_max_) <1.9 m/s as normal, 1.9–2.4 m/s as equivocal (unknown if affected or unaffected) and >2.4 m/s as SAS affected (www.archcertify.org). For this study, subvalvular aortic stenosis cases were defined by presence of a left-basilar systolic heart murmur and a maximal aortic outflow tract velocity exceeding 2.5 m/s. Controls were defined by having a maximal aortic outflow tract velocity ≤1.8 m/s. Dogs with maximal aortic outflow tract velocities ranging from 1.81 to 2.49 were classified as equivocal for SAS. Animals were excluded from the study if any other congenital heart disease was observed by routine two-dimension, m-mode, color and spectral Doppler echocardiogram.

Pedigree analysis was performed on an extended family of 45 Newfoundlands. A pedigree was constructed to evaluate the pattern of inheritance. These dogs were not utilized in the genotype analysis due to close family relationship.

Whole venous blood was obtained in ethylenediaminetetraacetic acid (EDTA) for each affected and control dog identified for genetic analysis. Genomic DNA samples were prepared (Meurs et al. 2000). In short, cells were osmotically lysed in 2X sucrose-Triton (pH 7.6), Tris–NH_4_Cl buffer (pH 7.2) and nuclei were pelleted by centrifugation (2,000 rpm for 20 min at 25 °C). Pellets were resuspended in Saline-EDTA (pH 8.0) with 20 % SDS and proteinase K, and incubated overnight at 56 °C. The samples were subjected to phenol:chloroform:isoamyl (25:24:1, pH 8) and one chloroform extraction. Finally, the DNA was precipitated (95–100 % ethanol and 2 M NaCl), and resuspended in 100–200 μl of TE buffer (10 mM Tris, 1 mM EDTA, pH 8).

Samples of DNA from 24 cases and 24 control Newfoundlands that were unrelated based upon three generation pedigrees were selected for genotyping performed by GeneSeek Agrigrenomics and Veterinary Diagnostics^1^ using the CanineHD BeadChip^2^ to obtain single-nucleotide polymorphism (SNP) data for >170,000 SNPs in the canine genome. Dogs classified as equivocal were excluded from SNP analysis due to uncertainty of disease status.

Genotype quality control, filtering and analysis were performed using PLINK^3^ (Purcell et al. 2007). Genotype data was filtered to exclude individuals with >10 % missing genotypes and SNPs with low genotype success <0.03. SNPs with a minor allele frequency (MAF) <0.05 were excluded from the analysis. Data analysis was performed with a simple association approach. A simple association genome-wide suggestive criteria was set at *P* ≤ 5 × 10^−5^ (Wellcome Trust Case Control Consortium 2007). Odds ratios were obtained from the simple association in PLINK and standardized by inversion if less than one. Manhattan plots were constructed for the association data using R software.^4^

Two previously identified Newfoundland dogs with SAS were donated for sample collection post-mortem and the regions of subvalvular aortic stenosis and ridge tissue were harvested and flash frozen. Total RNA was routinely extracted from the subvalvular ridge utilizing the RNeasy Fibrous Tissue Mini Kit.^5^ Additionally, total RNA from six other non-Newfoundland dogs without subvalvular aortic stenosis was utilized for comparison and as a part of the ongoing genetic research for other disease processes.

Total RNA quality was checked by running a sample on the Bioanalyzer 2100 using the RNA 600 Nano Assay.^6^ Samples with a RIN score of 8 or higher were quantified on a Qubit 2.0 Fluorometer^7^ and libraries made using the TruSeq RNA sample preparation kit, V2^8^ following the manufacturer’s protocol. In short ERCC ExFold RNA Spike In Mix (see footnote 5) was added to total RNA and poly-A RNA was enriched using poly-oligo-T magnetic beads (see footnote 5). The enriched mRNA was fragmented at 98 °C and primed in the presence of Illumina’s Elute, Prime, Fragment Mix (see footnote 8). First-strand cDNA was made from the fragmented mRNA by priming with random hexamer primers and incubating with reverse transcriptase. Second-strand cDNA was synthesized and the double-stranded cDNA purified using AMPure XP magnetic beads.^9^ The cDNA fragments had ends repaired, A tails and adapters added, underwent PCR amplification and cleaning steps according to Illumina’s TruSeq protocol (see footnote 8).

The TruSeq libraries were initially assessed for quality by sequencing at a low level on Illumina’s HiSeq system (see footnote 8). Specifically, 24 libraries were multiplexed per flowcell lane and a 36 bp run performed according to Illumina’s protocol (see footnote 8). The resulting sequences were assessed for quality and mapped to the CanFam3 genome to confirm insert content. Based on tag numbers from the 36 bp sequence run libraries were each loaded on one lane of an Illumina (see footnote 8) flowcell and 76 bp sequenced from both ends of the inserts. High-quality sequence reads passing the Illumina purity filter (see footnote 8) were utilized for further analysis of variants after their adapter sequences were clipped using FastqMcf.^10^

Reads were aligned to canFam3 genome using TopHat (v1.3.3).^11^ All possible PCR duplicate reads were identified using Picard MarkDuplicates.^12^ Multi-sample variant calling was performed using Samtools^13^ and variants were filtered to exclude known SNPs from dbSNP version 131^14^ (sourced from UCSC genome browser for canFam2, mapped to canFam3 using UCSC’s liftOver tool,^15^ then subtracted from the called variants using bedtools).^16^ Variants were then filtered with a custom Perl^17^ script requiring the non-reference variant to be present in at least one Newfoundland dog sample but not in any of the other non-Newfoundland dog samples. All variants were entered into Variant Effect Predictor (VEP Ensembl v. 70)^18^ and reported. Variants identified on the chromosomal regions of interest previously identified by GWAS were reviewed and reported if present in both affected Newfoundland dogs.

Two additionally affected Newfoundland dog DNA samples were used for preliminary validation screening of identified variants. Primers were designed using Primer3 and variants were validated by standard PCR amplifications carried out using AccuPrime GC-rich buffer A (see footnote 5), 2 unit/μl AccuPrime GC-rich DNA polymerase (see footnote 5), 20 mM each amplification primer, and approximately 100 ng of template DNA. Samples were denatured for 3 min (min) at 95 °C followed by 30 cycles of 95 °C for 30 s (s), 60 °C for 30 s, 72 °C for 30 s, and finally 72 °C for 10 min. The annealing temperature was optimized (55–60 °C) to accommodate the respective primer.

Residual amplification primers and dNTPs were removed from the PCR product using a single-step enzymatic cleanup kit.^19^ Amplicons were then subjected to nucleotide sequence determination and analyzed on ABI 3730XL sequencer.^20^ The nucleotide sequences were initially evaluated for a sequence change between affected dogs and the published normal canine sequence, (http://genome.ucsc.edu/cgi-bin/hgc?hgsid=322959945&c=chr18&o=46555041&t=46830526&g=xenoRefGene&i=NM_000218) to see if the variants matched those reported by RNA sequencing data. Variants passing this phase of quality control were then investigated in a large cohort of dogs to include 26 affected Newfoundland dogs, 23 non-affected Newfoundland dogs and 180 control dogs of 30 different breeds.

Statistical analysis of association was performed using Prism.^21^ A fisher’s exact test was utilized to report association within the Newfoundland dog study population. Effect size was determined through calculation of Relative risk and odds ratio. The sensitivity, specificity and mutation penetrance were calculated.

The normal and altered sequences of significantly associated variants were evaluated for changes that occur in the secondary structure or function with five software programs that assess the impact of genetic variants in various ways. These include, GORIV^22^ (http://npsapbil.ibcp.fr/cgibin/npsa_automat.pl?page=npsa_gor4.html; Combet et al. 2000), Provean^23^ v1.1 (http://provean.jcvi.org/about.php; Chio et al. 2012), Mutation Taster^24^ (http://www.mutationtaster.org; Flicek et al. 2012), I-TASSER^25^ server (http://zhanglab.ccmb.med.umich.edu/I-TASSER/; Zhang 2008) and Protean 3D^26^ (DNAStar, Madison, WI, USA). GORIV (see footnote 22) predicts the probability of a secondary structure at each amino acid position (Combet et al. 2000). Mutation Taster (see footnote 24) evaluated disease-causing potential of genetic sequence alterations (Flicek et al. 2012). Provean (see footnote 23) predicts tolerance for mutations and scores them as deleterious or not deleterious (Chio et al. 2012). I-TASSER (see footnote 24) predicts the effect of a given amino acid change on the protein structure in a three-dimensional model (Zhang 2008). Commercial software (Protean 3D)(see footnote 26) was used to further analyze the I-TASSER (see footnote 25) output files.

Protein products from the most highly associated variant, a 3 base insertion in PICALM, were further investigated through immunohistochemical evaluation. Myocardial sections of the LVOT from the university pathology repository from two SAS-affected Newfoundland dogs (one heterozygous for the PICALM insertion and one homozygous for the PICALM insertion) and a normal beagle dog were utilized. Serial sections were first stained by routine hematoxylin and eosin, Masson’s trichrome, and Verhoeff-Van Gieson stains to provide histopathologic classification of the SAS lesions. PICALM immunohistochemistry was then carried out as follows: Paraffin-embedded tissue sections were subjected to an antigen retrieval protocol [95 °C for 20 min in 10 mmol/l citrate buffer (pH 6.0)] followed by incubation with anti-PICALM antibody (1:200; LS-B5065, Lifespan BioSciences Inc). A biotinylated goat anti-rabbit IgG was used as the secondary antibody. Detection was made by avidin–biotin complex kit (Vector Laboratories, Burlingame, CA) and 3,3′-diaminobenzidine as the chromagen (BioGenex, San Ramon, CA); Sections were counterstained with hematoxylin.

The role of PICALM in cardiac morphogenesis was investigated through the use of in vivo small molecule inhibition. PICALM functions as a clathrin recruiter to the cell membrane and as such changes in clathrin availability are a plausible downstream effect of mutations in PICALM (Maritzen et al. 2012). Therefore, a commercially available, cell-permeable clathrin inhibition molecule (PITSTOP 2 ab120687, Abcam, Cambridge, MA, USA) was applied to developing *X. laevis* embryos to evaluate effects on cardiac morphology and determine whether developmental changes similar to SAS may be observed as a result of inhibition of clathrin-mediated endocytosis.

*Xenopus laevis* embryos were obtained by in vitro fertilization as previously described, de-jellied with 2 % cysteine-HCl (pH 7.8–8.1), sorted to eliminate abnormal individuals, and cultured in 0.1 × MMR (Marc’s Modified Ringers; Sive et al. 1998) at 15–23 °C (Sive et al. 1998). Staging was according to standard methods (Nieuwkoop and Faber 1994). Stock solutions of Pitstop 2 (abcam #ab120687) were prepared in DMSO to a concentration of 30 mM, as suggested by product information. Four embryos were exposed in each well of a 12-well plate starting at stage 27/28 (Nieuwkoop and Faber 1994) after fertilization, at final concentrations of 1 and 5 mM. Control embryos were exposed to an equivalent volume of DMSO only.

Whole embryos were fixed after cardiogenesis as previously described (stage 40) for immunofluorescence staining for 45 min in 4 % paraformaldehyde solution (100 mM Hepes pH 7.4, 100 mM NaCl, 4 % paraformaldehyde), followed by four washes in −20 °C Dent’s fixative (80 % methanol/20 % DMSO) solution (Fagotto and Gumbiner 1994). After overnight storage, the embryos were rinsed three times with Tris–NaCl (100 mM Tris–HCl pH 7.3, 100 mM NaCl), then incubated for 1 h in Tris–NaCl. The Tris–NaCl solution was replaced with sucrose/gelatin (15 % sucrose/25 % cold water fish gelatin). After overnight equilibration, the embryos were embedded and frozen in O.C.T. compound (Tissue-Tek). Ten micron sections were cut on a Leica CM1850 cryostat and picked up on coated slides.

Slides were allowed to warm to room temperature, then post-fixed in acetone for 1 min before being air dried and blocked for 30 min in Blocking Buffer (Reed et al. 2009). For immunohistochemical staining, slides were incubated overnight at 4 °C as described (Reed et al. 2009) with blocking buffer containing the following primary antibody dilutions: Fibrillin (DSHB, JB3, 1:100), β-catenin (SCBT, H-102; 1:100). After primary antibody incubation, the slides were washed twice in PBT for 5 min. The slides were then incubated for 3 h in blocking buffer containing Alexa 488-conjugated goat anti-mouse IgG (Invitrogen, A11029; 1:2,000) and/or Alexa 546-conjugated goat anti-rabbit IgG (Invitrogen, A11035; 1:2,000), and washed twice in PBT for 5 min each. Autofluorescence was quenched in Eriochrome Black (0.2 % w/v in PBS, 5 min), followed by several washes in PBS. Slides were mounted in Prolong Gold (or Prolong Gold with DAPI), cured overnight in the dark, and then sealed with nail polish. Fluorescence was visualized on a Leica SPEII confocal microscope.

## Results

### Pedigree analysis

**Fig. 1:**
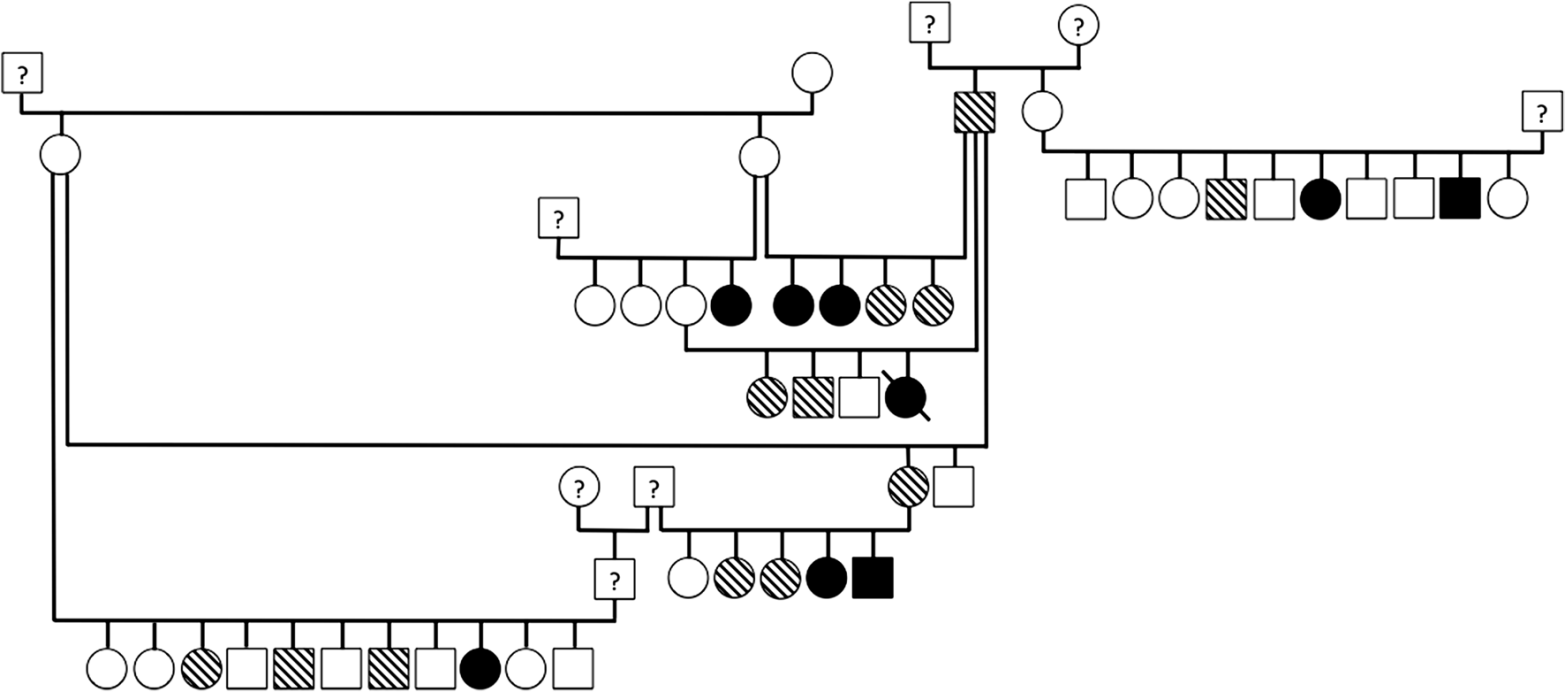
Pedigree representing an extended family of 53 Newfoundlands evaluated for SAS and depicted with the following key: *circles* represent females; *squares* represent males; *open symbols* are unaffected; *blackened* *symbols* are affected; *striped symbols* are equivocal; a *diagonal line* through the symbol represents sudden death; and symbols containing *question marks* have unknown phenotypes as they were not evaluated as part of this study

Pedigree analysis was performed on information obtained from a family of 45 Newfoundlands. Nine out of 45 dogs (7 females, 2 males) were found to be clearly affected with SAS and had a median *V*_max_ of 3.5 m/s (2.5–6.0), 12 (7 females, 5 males) were considered equivocal with high suspicion for SAS and had a median *V*_max_ of 2.0 m/s (1.9–2.3) and 24 were unaffected (14 females, 10 males) with a median *V*_max_ of 1.7 m/s (1.4–1.8). All equivocal and affected dogs within this extended family had a systolic heart murmur and were noted to have color-flow Doppler evidence of aortic insufficiency and/or a subvalvular ridge visible by 2D echocardiogram of the LVOT. Equivocal dogs when mated with normal dogs produced clearly affected puppies. Within this family, two separate matings of an SAS equivocal dog to a normal dog produced severely affected puppies with aortic velocities exceeding 5.0 m/s. No unaffected to unaffected mating was observed in this family, however, the presence of either an affected or equivocal dog within each generation supports the previous reports of a possible dominant pattern of inheritance with variable penetrance in Newfoundland dogs with SAS (Fig. 1).

### Genome-wide association study

A separate population of 48 Newfoundlands that were unrelated, at least within three generations, was used for the GWAS study. The genotyped control dogs (10 males, 14 females) had a median age of 3.5 years (1–8 years) and LVOT *V*_max_ of 1.6 m/s (1.5–1.75). Genotyped cases (11 males, 13 females) had a median age of 2.5 years (14 weeks–7 years) and LVOT *V*_max_ of 3.83 m/s (2.5–7.5).

**Fig. 2:**
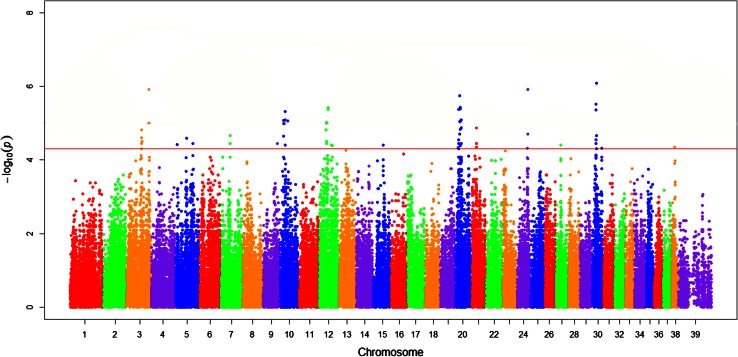
Manhattan plot demonstrating association of SNP markers with SAS in Newfoundlands graphed as chromosomal location versus –Log10 of *P*_raw_ value. The *red line* represents the threshold of genome-wide suggestive loci

After frequency and genotype pruning of individuals and SNPs, 18 of 24 cases and 20 of 24 controls remained and 122,184 out of 173,622 SNPs remained for analysis. Thirteen chromosomal regions met genome-wide suggestive threshold for association with disease phenotype as follows: chromosomes 3, 5, 7, 9, 10, 12, 15, 20, 21, 24, 27, 30 and 38 (Fig. 2). Genome inflation factor based upon a median Chi-squared test equaled 1.87 indicating a component of population stratification such as cryptic relatedness between the samples and potentially explaining the multiple chromosomal regions of interest (Yang et al. 2011).

### RNA sequence analysis

**Fig. 3:**
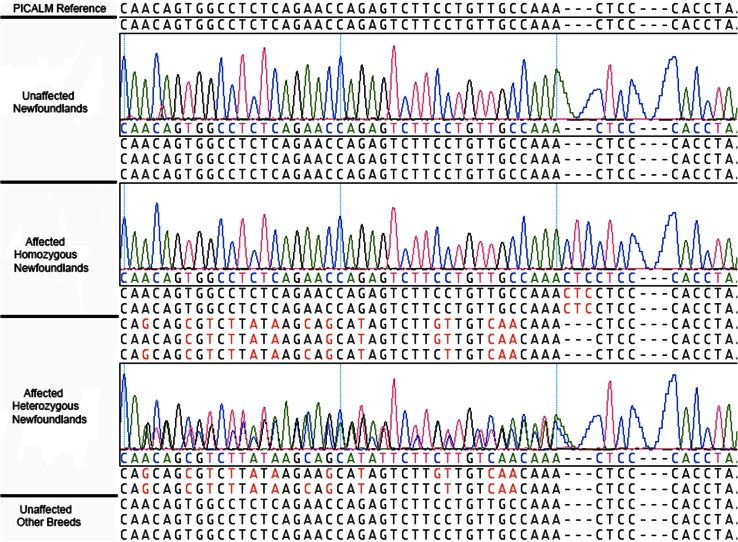
Comparative sequencing results showing unaffected Newfoundland dogs, homozygous mutant Newfoundland dogs, heterozygous mutant Newfoundland dogs and other unaffected dog breeds as sequencing files with expanded tracings for comparison

**Fig. 4:**
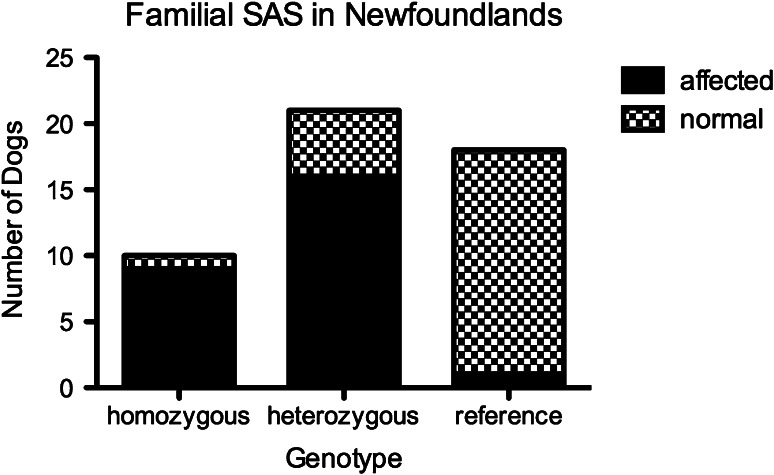
A *bar graph* represents genotypes of Newfoundland dogs sequenced for the PICALM insertion associated with SAS. *Checkered regions* represent normal Newfoundland dogs while *blackened* regions demonstrate SAS-affected Newfoundland dogs

RNA sequence analysis revealed a three-nucleotide insertion in exon 16 of canine PICALM at amino acid position 600 (K599_L600insL). The insertion adds a leucine to the amino acid sequence (Fig. 3). Validation of this variant was performed and found to associate with disease by Fisher’s exact test (*P* < 0.0001). The odds ratio was estimated at 70.83 (95 % CI 7.8–642) with a relative risk of 14.52 (95 % CI 2.14–98.32). Sensitivity and specificity for disease prediction based upon mutation status were 96 and 74 %, respectively. Overall 25 of 26 SAS-affected Newfoundland dogs possessed the insertion mutation (9 homozygous, 16 heterozygous); 6 of 23 non-affected Newfoundland dogs possessed the mutation (1 homozygous, 5 heterozygous); and none of 180 control dogs of 30 breeds possessed the mutation in any form (Fig. 4). Penetrance is thus calculated within this study population at 80.6 % in Newfoundland dogs and at 76 and 90 %, respectively, for heterozygous and homozygous genotypes. Three additional non-newfoundland dogs with SAS were evaluated and found to possess the same PICALM insertion (1 German Shepherd Dog, 1 American Staffordshire Terrier and 1 golden retriever dog).

Provean (see footnote 23) prediction revealed the K599_L600insL variant to be deleterious with a Provean score of −3.797 (Chio et al. 2012). GorIV (see footnote 22) secondary structure analysis of canine PICALM exon 16 mutant and normal sequences predicted reduction in the extended strand downstream from the insertion and increased random coil in the mutant protein product (Combet et al. 2000).

**Fig. 5:**
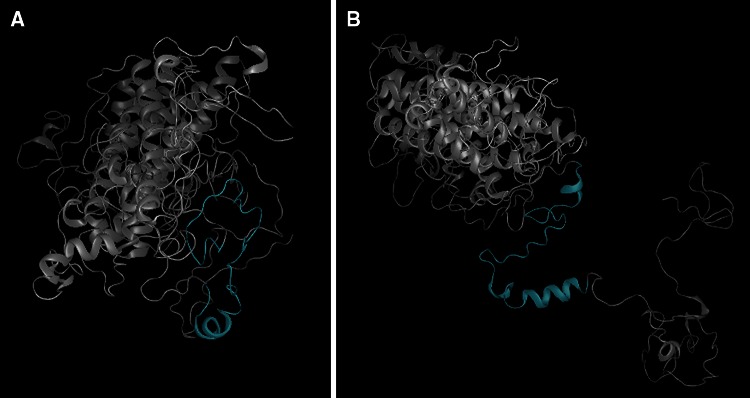
Three-dimensional protein models of the PICALM gene in normal dogs (**a**) and mutant Newfoundland dogs (**b**) with the 599K_600LinsL mutation. The downstream protein conformation is predicted as distorted. The *blue shaded* region on each protein model corresponds to exon 16 where the insertion mutation is location in SAS-affected Newfoundland dogs

I-TASSER three-dimensional protein modeling identified visible structural variation that impacts the remainder of the protein (see footnote 25) product downstream from the insertion mutation (Fig. 5; Zhang 2008). Mutation Taster (see footnote 23) predicts possible loss of a function protein region due to the insertion mutation centered on a possible loss of breakpoint for translocation to form CALM/MLLT10 fusion protein a transcriptional regulation complex with a confidence for accurate predictions at 0.99/1.0 (Flicek et al. 2012).

### Immunohistochemical analysis

**Fig. 6:**
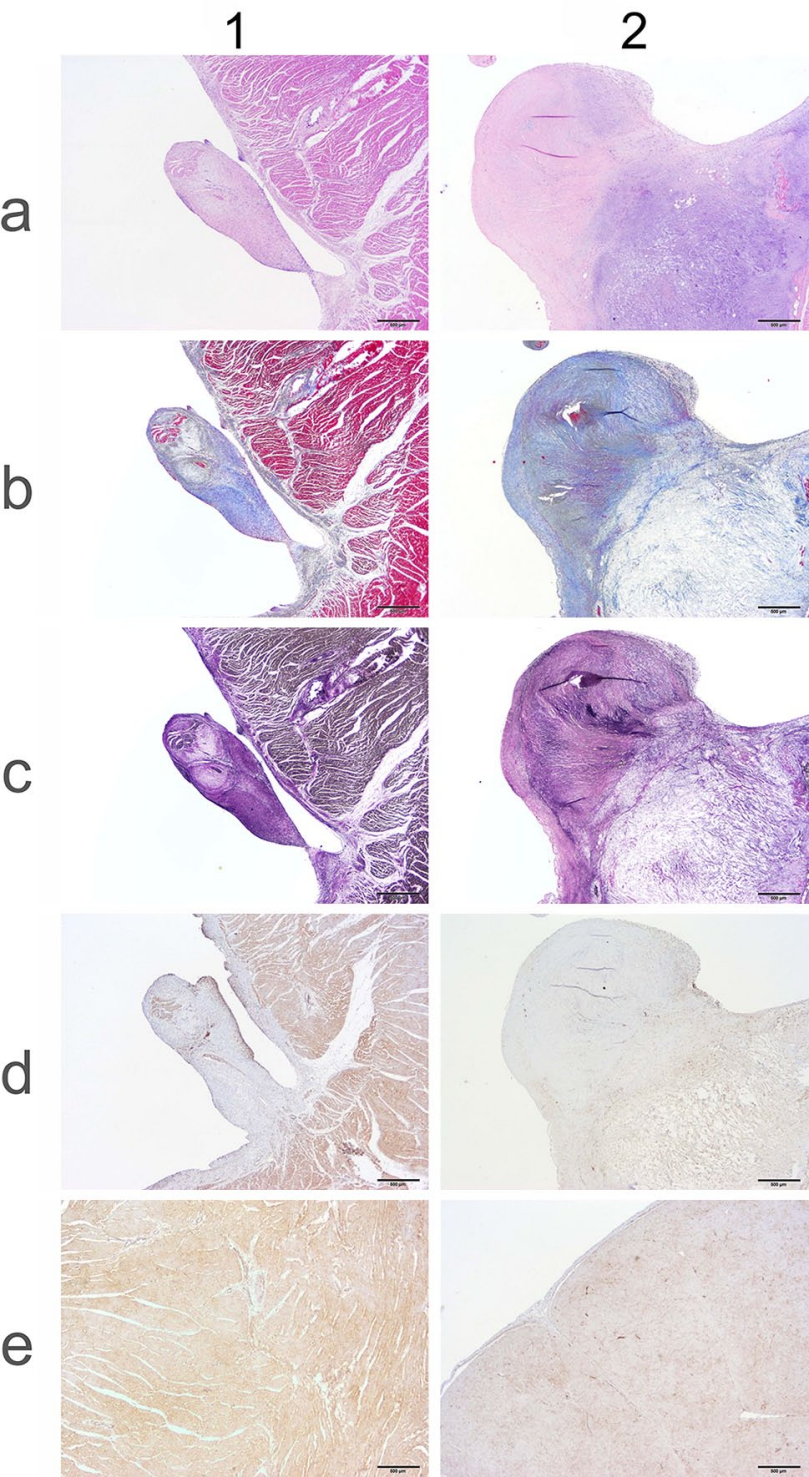
Comparative histopathology of a LVOT Subvalvular ridge and myocardium from a Newfoundland heterozygous for the PICALM insertion (*1*) and a Newfoundland homozygous for the PICALM insertion (*2*). Light microscopy images at 20× resolution are presented for routine hematoxylin and eosin (**a**), Masson’s trichrome (**b**), Verhoeff-Van Gieson (**c**) staining for elastic tissue *black* in color, PICALM antibody in LVOT ridge (**d**) staining PICALM *brown* and PICALM antibody in LV myocardium (**e**) staining PICALM *brown*

All negative control slides revealed absence of background staining and appropriate quality control. The Subvalvular ridge region of the heterozygous Newfoundland and homozygous Newfoundland dogs were morphologically diverse. All samples contained obvious collagen and fibrous connective tissue as identified by Masson’s trichrome staining. VVG staining revealed small amounts of black elastic staining within the ridge and supporting myocardial tissue. PICALM antibody positivity was identified in the ridge and the myocardium of all dogs including the myocardium of the control beagle, demonstrating its presence in myocardium and LVOT. The appearance of the ridge and its immunohistochemical staining pattern were diverse between the two genotypes, however, sample numbers are not sufficient to identify differences in protein expression by genotype beyond confirmation of PICALM presence in these tissues (Fig. 6).

### Small molecule inhibition of clathrin

**Fig. 7:**
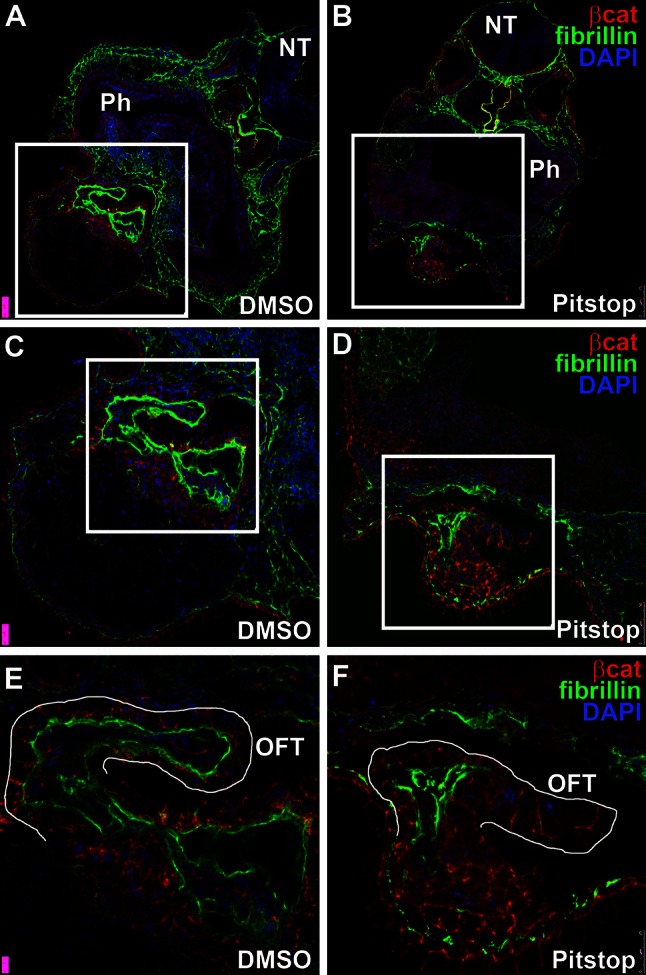
Comparative immunohistochemical imaging of a stage 40 *Xenopus laevis* control embryo (DMSO) versus a 5 uM treated Pitstop embryo. The *top row* **a**, **b** shows the full section while the *middle row* **c**, **d** and *bottom row* **e**, **f** are successive magnification of the squared region to better visualize the outflow tract. The neural tube (NT), outflow tract (OFT), and pharyngeal cavity (Ph) are visualized. The fluorescent staining represents fibrillin (*green*), beta-catenin (*red*) and DAPI nuclear stain (*blue*)

Small molecule inhibition of clathrin resulted in developmental abnormalities in stage 40 *X. laevis* embryos as depicted (Fig. 7). Clathrin-inhibited embryos exhibited stenotic and shortened cardiac outflow tracts when compared to control embryos. These in vivo pharmacological inhibition studies are consistent with a functional role for PICALM in outflow tract morphogenesis.

## Discussion

Subvalvular aortic stenosis is a complex and significant congenital heart disease observed in both dogs (Buchanan 1999; Tidholm 1997) and human beings (Valeske et al. 2011). Although it is often characterized by the presence of an abnormal ridge of tissue in the left ventricular outflow tract, the morphology of the lesion can be varied and include ring or diffuse tunnel lesions (Pyle and Patterson 1976; Jones et al. 1982; Valeske et al. 2011). The familial and molecular basis of the defect in both species is poorly understood although a previous description of the pattern of inheritance in a canine model in the Newfoundland dog breed suggested either an autosomal dominant with incomplete penetrance or a polygenic mode of inheritance (Pyle and Patterson 1976). The findings reported here in this same canine model are most closely suggestive of an autosomal dominant mode of inheritance with incomplete penetrance. Interestingly, the results are particularly consistent with this mode of inheritance if dogs classically phenotyped as equivocal due to weak phenotypic findings are reclassified as affected. In the family provided here, several affected dogs were produced from the mating of an unaffected and an equivocal dog. Importantly, although this mode of inheritance is plausible, it is equally plausible to consider more complex inheritance patterns for this condition. The worsening of SAS lesions with advancing age makes phenotypic characterization of this disease challenging in young dogs. The wide variety of lesions associated with condition further confounds diagnosis in the antemortem period. Understanding this combination of concerns is crucial to pedigree analysis. As such, an intermediate mode of inheritance with or without complete penetrance must be considered for this disease and cannot be excluded based upon these Newfoundland dog families.

Molecular analysis identified that a genetic mutation in an exonic region of the PICALM gene was strongly associated with the development of SAS in the dogs evaluated here. Although a single region of interest was not readily identified by genome-wide association study, genome-wide suggestive regions were found which highlighted the chromosome harboring the identified mutation in PICALM. This underscores a limitation of GWAS in a purebred dog population where individuals are frequently related and the population substructure may be poorly understood.

PICALM is a known mediator of endocytosis at the cellular level and this process is involved in the morphogenesis of the heart during embryonic development. PICALM is involved in cellular trafficking, endocytosis regulation and clathrin-mediated vesicle formation (Maritzen et al. 2012). It is tightly associated with iron homeostasis and cellular proliferation (Scotland et al. 2012). These physiologic roles are strongly tied to embryonic morphogenetic events, including those that occur in the neural crest and heart (Strilic et al. 2010). Since PICALM functions as a clathrin recruiter (Maritzen et al. 2012), the role of PICALM in cardiac morphogenesis was investigated in *X. laevis* embryos by inhibiting clathrin to demonstrate the likely impact of a PICALM mutation. The embryos demonstrated outflow tract changes when compared to the control embryos. Aggregation of amyloid substance is one function of PICALM and may represent a pathogenic mechanism of neural plaque formation and we propose a similar aggregation mechanism as one possibility for generation of the subvalvular ridge in SAS (D’Angelo et al. 2013). While the exact functional mechanism for generation of the subvalvular ridge in SAS is yet to be determined, a role for PICALM in cardiac morphogenesis is strongly suggested by our experiments with *X. laevis* embryos in which inhibition of clathrin-mediated endocytosis demonstrated the likely impact of a PICALM mutation.

This study represents the first report of mutation association for SAS in dogs. Molecular analysis identified that a genetic mutation in an exonic region of the PICALM gene was strongly associated with the development of SAS in this canine model as well as in three other dogs from different breeds with SAS, indicating that the PICALM insertion is not isolated to that one breed and may be important in other breeds and species as well. Future investigation into other breeds with SAS and human beings that have an inherited form of discrete subaortic stenosis is warranted.

## Footnotes

1 GeneSeek Agrigrenomics and Veterinary Diagnostics, Lincoln, Nebraska, USA.

2 Illumina CanineHD BeadChip, Illumina, San Diego, California, USA.

3 Plink, Shaun Purcell, http://pngu.mgh.harvard.edu/purcell/plink/.

4 The R project for statistical computing, version 2.15.3, http://www.r-project.org.

5 Qiagen Sciences, Germantown, Maryland, UAS.

6 Agilent Technologies Inc, Santa Clara, California, USA.

7 Life Technologies, Grand Island, New York, USA.

8 Illumina, San Diego, California, USA.

9 Beckman Coulter Genomics, Danvers, Massachusetts, USA.

10 Fastq MCF, http://code.google.com/p/ea-utils/downloads/list.

11 Tophat v1.3.3, http://tophat.cbcb.umd.edu.

12 Picard, http://picard.sourceforge.net/command-line-overview.shtml.

13 Samtools, http://samtools.sourceforge.net.

14 DbSNP v 133, http://www.ncbi.nlm.nih.gov/projects/SNP/.

15 UCSC LiftOver, http://genome.ucsc.edu/util.html.

16 Bedtools, http://code.google.com/p/bedtools/.

17 Perl, http://www.perl.org.

18 Variant Effect Predictor, v.70, http://useast.ensembl.org/info/docs/variation/vep/index.html

19 ExoSAP-IT, Affymetrix, Santa Clara, CA, USA.

20 Applied Biosystems, Foster City, CA, USA.

21 Prism 5, GraphPad Software Inc, La Jolla, California, USA.

22 GORIV, http://npsapbil.ibcp.fr/cgibin/npsa_automat.pl?page=npsa_gor4.html; Combet et al. 2000.

23 Provean v1.1, http://provean.jcvi.org/about.php; Chio et al. (2012).

24 Mutation Taster, http://www.mutationtaster.org; Flicek et al. (2012).

25 I-TASSER server, http://zhanglab.ccmb.med.umich.edu/I-TASSER/; Zhang (2008).

26 Protean 3D, DNAStar, Madison, WI, USA.
